# Enteroviral Meningitis-Associated Respiratory Deterioration in a Neonate Treated With High-Flow Nasal Cannula Therapy

**DOI:** 10.7759/cureus.102179

**Published:** 2026-01-23

**Authors:** Rei Sato, Takuma Ohnishi, Mizue Tomita, Maki Nakazawa, Isamu Kamimaki

**Affiliations:** 1 Department of Pediatrics, National Hospital Organization (NHO) Saitama Hospital, Wako, JPN

**Keywords:** apnea, aseptic meningitis, coxsackievirus, enteroviral meningitis, high-flow nasal cannula

## Abstract

Enteroviral meningitis is a common cause of aseptic meningitis in infants, yet respiratory deterioration, including central hypoventilation, is seldom emphasized. High-flow nasal cannula (HFNC) therapy may offer benefits beyond oxygen delivery. We report a case of a six-day-old term boy with enteroviral meningitis caused by coxsackievirus B2. He developed intermittent oxygen desaturation, bradypnea, and hypercapnia that were unresponsive to standard oxygen supplementation. HFNC rapidly stabilized his respiratory rate and oxygen saturation. This case suggests that HFNC may be a useful noninvasive option for central hypoventilation in viral meningitis. Its potential mechanisms include providing mild positive airway pressure, reducing dead space, and influencing central respiratory drive. Early recognition and timely initiation of HFNC could help prevent intubation and mechanical ventilation in similar cases.

## Introduction

Enteroviruses account for approximately 85% of cases of aseptic meningitis [1]. While common clinical presentations include fever, irritability, and gastrointestinal symptoms, respiratory deterioration, including central hypoventilation, is a clinically important yet potentially underrecognized manifestation, particularly in neonates. In the autumn of 2024, an outbreak of enteroviral meningitis occurred in Japan [2]. During this period, we encountered a six-day-old term boy with enteroviral meningitis who developed respiratory deterioration that improved with high-flow nasal cannula (HFNC) therapy.

## Case presentation

The patient was a six-day-old term boy born via uncomplicated vaginal delivery with a birth weight of 3,162 g. He remained well until day 6 of life, when he developed a fever and was transferred to our hospital. There were no known sick contacts. On admission to the pediatric ward, his body temperature was 37.9°C, and oxygen saturation (SpO₂) was 88% with 1 L/min of supplemental oxygen. His respiratory rate was 40 breaths per minute. He appeared alert and active. Physical examination was unremarkable, and lung auscultation was clear.

Laboratory testing showed normal C-reactive protein levels. Polymerase chain reaction (PCR) for SARS-CoV-2 and rapid antigen tests for respiratory syncytial virus, human metapneumovirus, and influenza using a nasopharyngeal swab were negative. Cerebrospinal fluid (CSF) analysis revealed pleocytosis with mononuclear predominance (Table 1).

**Table 1:** Laboratory analysis on admission pH: potential of hydrogen, pCO₂: partial pressure of carbon dioxide, HCO₃: bicarbonate or hydrogen carbonate, CSF: cerebrospinal fluid

Parameters	Patient value	Reference value [3]
Complete blood count		
White blood cells (x10^3^/µL)	13.3	9.1-34.0
Neutrophils “bands” (%)	4	3-5
Neutrophils “segs” (%)	44	54-62
Lymphocytes (%)	40	25-33
Hemoglobin (g/dL)	17.6	15.0-24.0
Hematocrit (%)	52.8	44-70
Serum electrolytes		
Sodium (mmol/L)	143	133-146
Potassium (mmol/L)	5.6	3.2-5.5
Chloride (mmol/L)	105	97-110
Blood chemistry		
C-reactive protein (mg/dL)	0.26	0.08-1.58
Albumin (g/dL)	3.6	2.5-3.4
Aspartate aminotransferase (U/L)	44	30-100
Alanine aminotransferase (U/L)	14	6-40
Lactate dehydrogenase (U/L)	380	170-580
Blood urea nitrogen (mg/dL)	6.6	3-12
Venous blood gas		
pH	7.425	7.35-7.45
pCO₂ (mmHg)	38.6	27-40
HCO₃ (mmol/L)	24.8	22-29
CSF analysis		
White blood cell (/µL)	181	<5
Mononuclear cells (%)	90	≥75%
Protein (mg/dL)	61	20-45
Glucose (mg/dL)	41	>50 (or 75% serum glucose)
Urinalysis (qualitative examination)		
Protein	Negative	Negative
Glucose	Negative	Negative
Urine sediments		
Red blood cell (/high power field)	<1	≤5
White blood cell (/high power field)	1-4	≤5

Chest and abdominal radiographs were unremarkable. Empirical therapy with acyclovir (60 mg/kg/day), cefotaxime (150 mg/kg/day), and ampicillin (300 mg/kg/day) was initiated. Oxygen therapy was discontinued shortly after admission because the patient maintained an SpO₂ of 98% on room air.

On the second day of illness, the patient experienced intermittent desaturation episodes requiring stimulation. SpO₂ dropped to the high 80 s but improved with supplemental oxygen and shoulder roll positioning. Subsequently, he developed supraclavicular retractions during inspiration. Although blow-by oxygen at 3 L/min stabilized SpO₂ at 99%, the retractions worsened. His respiratory rate fluctuated from a baseline of 30-40 breaths per minute down to 10-20 breaths per minute with intermittent episodes of tachypnea (60-70 breaths per minute). However, no distinct apneic episodes were observed. SpO₂ again dropped to approximately 90%. A repeat chest radiograph remained clear. Venous blood gas analysis revealed CO₂ retention (pCO₂ 57.7 mmHg from 38.6 mmHg at admission), suggesting hypoventilation. Capnography and arterial blood gas analysis were not performed. Given the worsening work of breathing despite blow-by oxygen, escalation of respiratory support was necessary. To avoid agitation often associated with face masks in infants, HFNC therapy (flow rate: 6-7.5 L/min; FiO₂​​​​​: 0.25) was initiated. Following initiation of HFNC, his respiratory rate stabilized without further episodes of bradypnea or tachypnea, and SpO₂ remained in the high 90 s.

**Figure 1:**
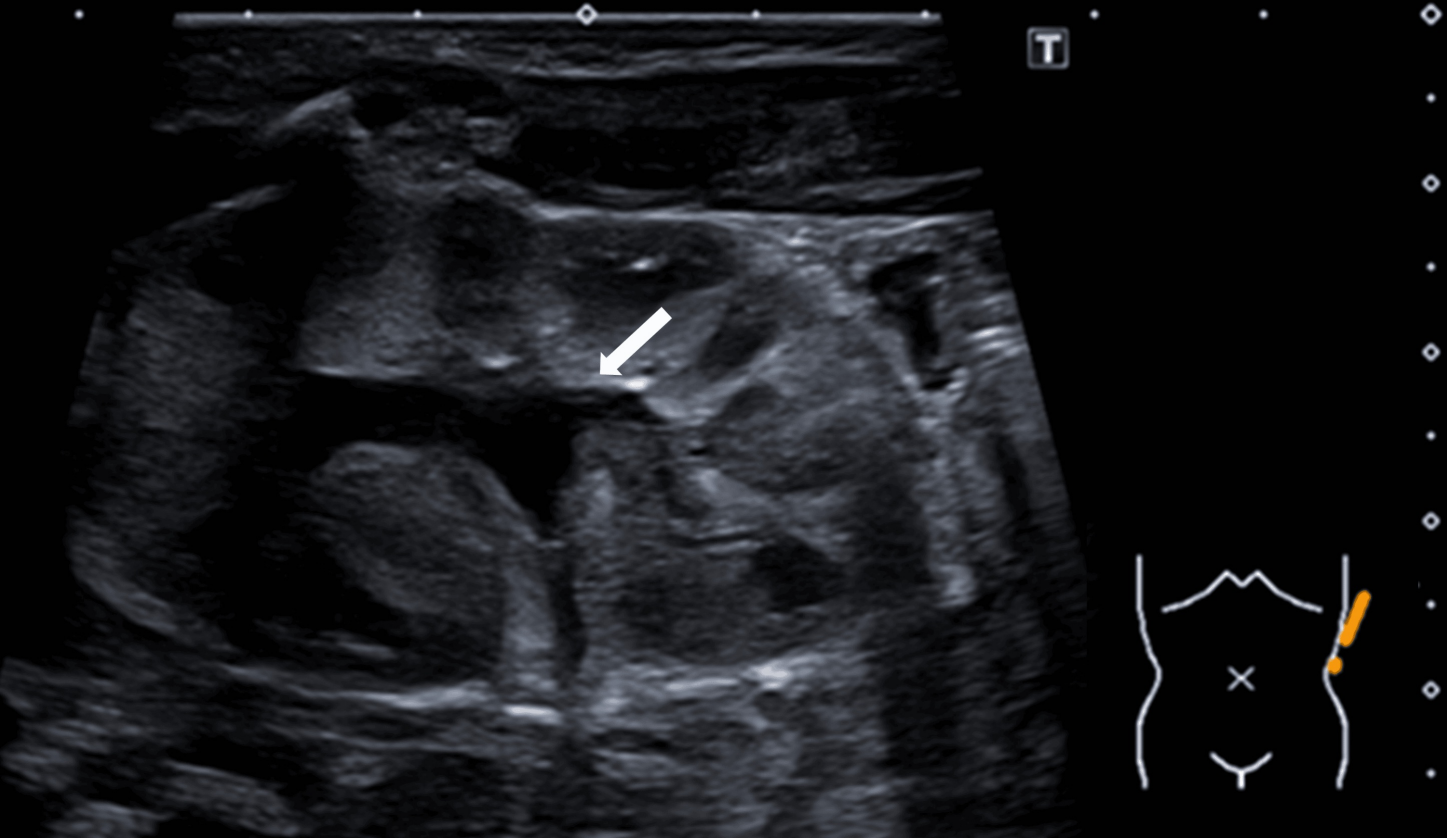
Abdominal ultrasound demonstrating grade 1 hydronephrosis of the left kidney

The BIOFIRE® FILMARRAY® Meningitis/Encephalitis Panel (BioFire Diagnostics, LLC, Salt Lake City, UT, USA) performed on CSF detected enterovirus, while CSF culture was negative. Blood cultures were also negative. Urinalysis of a catheterized specimen at admission showed no pyuria and a negative nitrite test (Table 1), and urine culture grew two pathogens: *Enterococcus faecalis* and *Escherichia coli* (10⁵ CFU/mL). Abdominal ultrasound demonstrated grade 1 left kidney hydronephrosis (Figure 1).

The patient became afebrile and was weaned off oxygen support on day 4 of illness. His respiratory status remained stable thereafter. Acyclovir and cefotaxime were discontinued on day 4 after confirming negative CSF culture results and the absence of herpesviruses on the multiplex PCR panel. Ampicillin was continued until day 9, and he was discharged on day 10. Additional PCR testing of the CSF identified coxsackievirus B2.

## Discussion

We report a case of a neonate with respiratory deterioration associated with enteroviral meningitis that was successfully managed with HFNC therapy. Previous reports have shown that increasing flow rates in HFNC therapy can reduce respiratory effort in children [4]. In neonates, HFNC may also help regulate respiratory rhythm by stimulating physiological vagal reflexes, thereby improving central hypoventilation [5].

In our patient, the respiratory deterioration appeared to have both obstructive and central components. Early in the course, the presence of supraclavicular retractions and improvement with positional change suggested an element of upper airway obstruction, possibly due to reduced pharyngeal tone. Subsequently, marked bradypnea, intermittent tachypnea, and hypercapnia without compensatory sustained tachypnea suggested central hypoventilation, possibly related to brainstem involvement from meningitis. The respiratory center normally adjusts ventilation in response to changes in blood CO₂ levels via chemoreceptor input; in this case, the administration of blow-by oxygen at 3 L/min was associated with CO₂ retention and no effective compensatory increase in respiratory rate, further supporting the diagnosis of central hypoventilation. The subsequent initiation of HFNC stabilized his respiratory rate and oxygen saturation. This effect may have been achieved not only through mild positive airway pressure and a reduction in anatomical dead space but also through modulation of central respiratory drive. A previous case report described the successful use of HFNC in a patient with apnea caused by parechovirus meningitis [5]. HFNC therapy could, therefore, represent a valuable noninvasive option for managing respiratory deterioration in meningitis, potentially avoiding the need for continuous positive airway pressure (CPAP) or intubation. However, careful monitoring is essential. In this case, we planned to escalate treatment to CPAP or intubation if HFNC failed. Based on established criteria [6], we defined treatment failure as (1) respiratory rate increasing from baseline or (2) an oxygen requirement exceeding an FiO₂ of 0.4 to maintain SpO₂ ≥92%.

HFNC is a widely used noninvasive respiratory support modality in pediatric patients. In a previous study comparing HFNC with nasal CPAP in preterm infants with apnea, no significant differences were found in the incidence or duration of apnea, bradycardia, or oxygen desaturation events between the two groups [7]. Moreover, HFNC was not associated with adverse effects such as mucosal dryness or nasal trauma. Further research is needed to clarify the effects of HFNC on respiratory effort in pediatric patients and to elucidate its underlying mechanisms.

Although enteroviral meningitis usually follows a mild course, severe cases requiring oxygen support can occur in early infancy. Reported risk factors include the absence of maternally derived neutralizing antibodies against the infecting serotype, maternal illness prior to or during delivery, prematurity, onset within the first few days of life, and infection with certain serotypes such as group B coxsackieviruses and echovirus 11 [8]. In our case, the onset occurred on day 6 of life, and the causative serotype, coxsackievirus B2, is known to be associated with severe disease, which may have contributed to the observed clinical severity.

Although bacteria were detected in the urine culture, we considered the possibility of contamination. This assessment was based on three findings. First, the urinalysis showed no leukocyturia. Second, aseptic meningitis was considered the primary illness and the most likely cause of fever. Third, two different bacterial species (*Enterococcus faecalis* and *Escherichia coli*) were isolated, which increases the likelihood of contamination. However, because the patient had grade 1 hydronephrosis, we remained concerned about the possibility of a concurrent urinary tract infection. Therefore, we continued intravenous ampicillin for more than one week. A previous study of 1,629 febrile infants aged 1 to 60 days found urinary tract infections in 13.2% and aseptic meningitis in 8.8%, but both infections occurred together in only 0.7% of cases [9]. While the likelihood of a concurrent urinary tract infection with aseptic meningitis is low, it cannot be completely excluded in this patient, and a potential contribution of bacterial infection to the respiratory deterioration cannot be definitively ruled out.

This report has several limitations. First, the diagnosis of central hypoventilation was primarily based on clinical observations and intermittent venous blood gas analyses. We did not utilize continuous physiological monitoring, such as capnography, to fully characterize the respiratory deterioration. Second, we were unable to record the exact frequency of desaturation episodes because they occurred frequently prior to the initiation of HFNC therapy. Finally, although rare, coinfection with both enteroviral meningitis and a urinary tract infection cannot be completely excluded. Therefore, the potential contribution of a bacterial infection to the respiratory deterioration remains unclear.

## Conclusions

We describe a case of a six-day-old term boy with enteroviral meningitis who developed respiratory deterioration that was successfully managed with HFNC therapy. This case highlights the potential utility of HFNC as a noninvasive option for respiratory support in viral meningitis. Further research is warranted to clarify its efficacy in this population. Clinicians should recognize that severe respiratory complications can occur in neonatal enteroviral meningitis and must initiate timely, appropriate therapeutic interventions.
